# Development of UPLC-MS/MS method and its pharmacokinetic application for estimation of sertraline in rat plasma

**DOI:** 10.1016/j.mex.2022.101750

**Published:** 2022-05-31

**Authors:** Mohammad Akhlaquer Rahman

**Affiliations:** Department of Pharmaceutics and Industrial Pharmacy, College of Pharmacy, Taif University, Taif, 21974, Kingdom of Saudi Arabia

**Keywords:** Development, Validation, Robustness, Pharmacokinetic application

## Abstract

The current research aims to develop a rapid, sensitive and robust UPLC-MS/MS method for quantitative estimation of sertraline in rat plasma following oral administration of lipid-based formulation. Different types of isocratic systems were tried for optimization of mobile phase to attain good resolution and appropriate retention time. The multiple reaction monitoring transitions of m/z 306.3 → 159.1 and 515.2 → 276.1 were used to quantify sertraline and internal standard, respectively. The method was validated for different parameters as per the guideline of the United States Food and Drug administration (USFDA). The validated linearity range of sertraline was found to be 1-1,00 ng/mL in rat plasma with 0.1 ng/mL as lower limit of quantification. The intra-day and inter-day precision (RSD%) were within 7.6-10.6% and the accuracies(RE%) were ± 8.0%. The results showed a good percentage recovery of analytes within acceptance limit. No significant degradation of drug in plasma was observed during the stability study. The method demonstrated short analytical run time (< 3 min), low sensitivity (0.1 ng/mL) and requirement of small amount of plasma (50 µL) for extraction procedure.

**Overall, this method**

• Results in an optimized and validated assay for estimation of sertraline in plasma.
• Offers a cost effective mobile phase with excellent linearity, sensitivity, accuracy, and precision. The method suggested its application in pharmacokinetic study of sertraline administered via oral route.
• The method can be used for therapeutic drug monitoring in drug designing. The method can also be used for toxicity and bioequivalence studies.

## Graphical abstract

**Figure:**
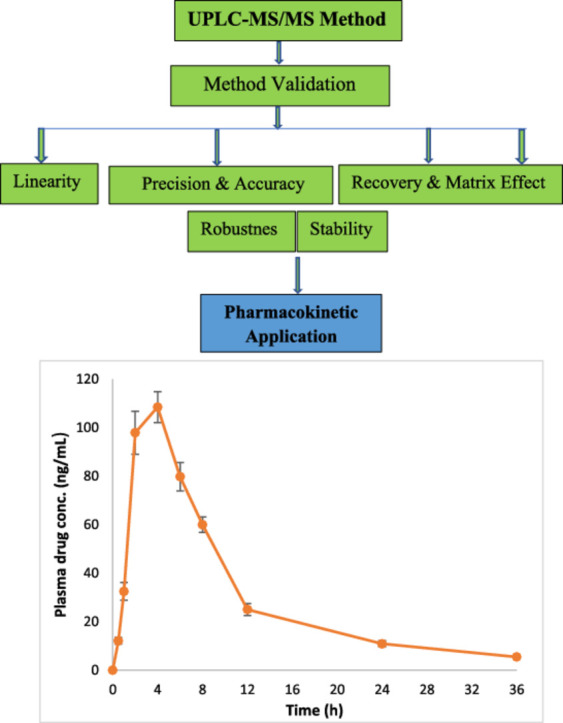

**Specifications table**

**Table:** 

Subject area:	Pharmacology, toxicology and pharmaceutical science
More specific subject area:	Analytical chemistry
Method name:	UPLC-MS/MS
Name and reference of original method:	N/A
Resource availability:	Reagents: Drug sertraline, purity >99.0% and Internal standard (IS) telmisartan, purity >99.5% (Ranbaxy, Gurgaon, India) Compritol® E ATO (Gattefosse, France) Tween® 80 (Merck India Ltd.) Acetonitrile and methanol, gradient grade (Sigma Aldrich, USA) EDTA disodium salt dehydrate (Acros Organics, USA) Instrument: Nexera UHPLC system (Waters Corp., USA) C18 column (Agilent technologies, USA) Electrospray ionization source (Waters Corp., USA) MassLynxV4.2 software (Waters Corp., USA)

## Background

Sertraline is prescribed as first line drug for major depressive disorders treatment. The drug belongs to the category of selective serotonin reuptake inhibitors. Reports unveiled that it is one of the most popular psychiatric medication in the US retail market [1]. The benefits of sertraline are due to its safely, tolerability and adverse effect profile [2]. Its oral absorption is very slow (4-8 h peak plasma concentration time) with 22-36 h elimination half-life [3]. In the past few years, different analytical techniques such as liquid chromatography [4], [5], [6], thin layer chromatography [7], tandem mass spectrometry [8,9], capillary electrophoresis [10] and gas chromatography–mass spectrometry [11,12] have been developed. However, most of the analytical methods resulted in certain limitations that include long run time of samples, low sensitivity, and requirement of large amount of plasma for the extraction procedures that constrain their application to high sample throughput. Above method demonstrated long analytical run time (>10 min), low sensitivity (≥ 5 ng/mL), and the requirement of a large amount of plasma (≥ 0.5 mL) for the extraction procedure. The lipid matrix may affect the extraction and analytical procedure, and no such report has been found for quantitative estimation of sertraline in such systems. Therefore, measurement of sertraline concentration in plasma following oral administration of lipid-based formulation was still required with a simpler and more economical mobile phase. In the present study, the ultra-performance liquid chromatography tandem-mass spectrometry (UPLC-MS/MS) has been developed. Furthermore, the method successfully applied in the pharmacokinetic studies of sertraline after oral administration of formulation in rats to provide a guidance for development and rational application of other drugs.

## Methods detail

### Chromatographic and mass spectrometric conditions

Nexera UHPLC system (Waters Corp., USA) assembled with a triple quadruple mass spectrometer (XEVO TQD) and an electrospray ionization source (Waters Corp., USA) was used for chromatographic method development. C18 column with internal diameter 2.1 mm × 50 mm, pore size 1.9 µm (Agilent technologies, USA) and inline 0.2 μm frit filter (Waters Corp., USA) were used for chromatographic separation. The column temperature was set to 40°C and autosampler temperature to 10°C. Methanol (A) and water (B) containing 0.1% glacial acetic acid (50/50, v/v) was used as the mobile phase system. Flow rate of mobile phase was set to 400 µL/min, the injection volume to 10 μL and the total run time to 2.5 min. The mass spectrometer was operated in MRM mode of transition (*m/z* 306.3 → 159.1 for the drug and 515.2 → 276.1 for IS). The source parameters optimized were as follows; curtain gas: 40 psig, nebulizer gas: 50 psig, turbo gas: 55 psig, ion spray voltage: 2.5 kV, and temperature to 450 ± 5°C. DaMassLynxV4.2 software (Waters Corp., USA) was used for the purpose of data acquisition and instrumental control.

### Preparation of calibration standard and quality control samples

A stock solution (0.1 mg/mL) was prepared by dissolving 10 mg of sertraline in 100 mL methanol. The above stock solution (1 mL) was again diluted with methanol (100 mL) to obtain working solution (1 µg/mL). The above working solution was further diluted with rat plasma to get different concentration levels (0.1, 2, 5, 10, 20, 50, 80, and 100 ng/mL). The samples were purified using membrane filter (pore size, 0.22 μm) before injection into the chromatographic system. Drug-plasma calibration standards were constructed after spiking the above standard calibration solutions (0.1-100 ng/mL) with rat plasma. The working solution (100 ng/mL) of IS was also prepared by dilution of the stock solution with methanol. Quality control (QC) samples at three different levels (5 as low, 25 as medium, and 100 ng/mL as high) were prepared in blank plasma for method validation.

### Method for extraction and sample preparation

The drug was extracted from rat plasma after collection of blood following oral administration of lipid-based formulation. Briefly, 100 μL of plasma sample, 100 μL of 0.1 M sodium hydroxide solution, and 1 mL ethyl acetate was placed into a 1.5 mL capacity centrifuge tube followed by vigorous shaking for complete mixing of all the contents. Then, the tube was centrifuged at 15,000 rpm for 10 min. The clear supernatant was taken out from the centrifuge tube. The drying was achieved using vacuum concentrator maintained at a temperature of 40°C. Further, 100 μL of mobile phase was added into the dried residue for reconstitution purpose. The reconstituted sample was again centrifuged for 5 min at the rate of 15,000 rpm. Finally, the clear supernatant was collected and stored at -20°C for analysis.

## Method validation

The guideline of United States Food and Drug administration (USFDA) was followed for the validation of the analytical method and the parameters taken into account were linearity, precision, accuracy, extraction recovery, matrix effect, robustness, and solution stability at different temperature conditions and time periods [13].

### Spectrometric and chromatographic conditions, optimization of mobile phase

**Fig. 1:**
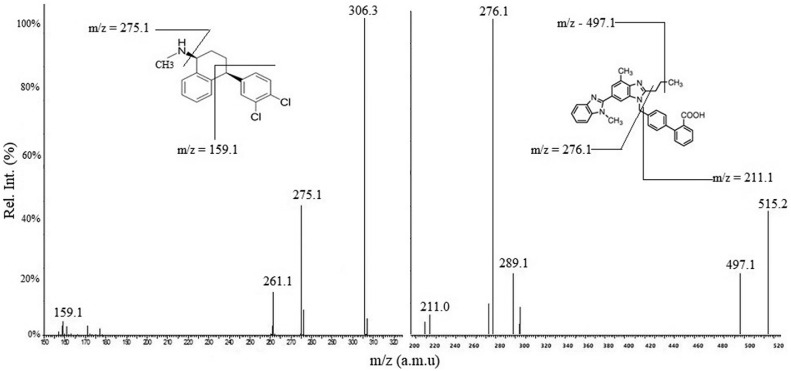
Full scan product ion spectra of parent ions and fragmentation pathways of (A) sertraline, molecular weight 306.3 and (B) Telmisartan, molecular weight 515.2 as in the positive mode of ion.

**Fig. 2:**
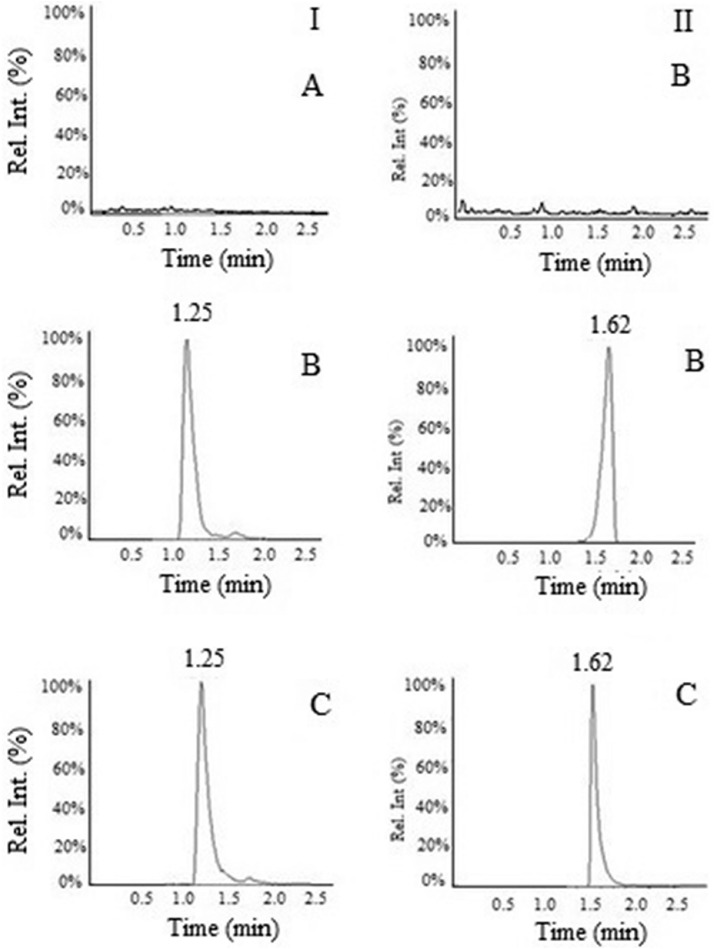
Representative chromatograms of blank plasma (A), the standard sample at LLOQ (B), and plasma sample after oral administration of formulation (C). Peak I, sertraline; peak II, telmisartan.

The most abundant daughter ions were obtained at m/z 159.1 for drug and 276.1 for IS. Fig. 1 represents the parent ion and fragment ions of drug and IS. Methanol-water and acetonitrile-water were tried as mobile phase to achieve good resolution and suitable retention time. Different proportion (0.1-0.5%) of glacial acetic acid, formic acid, and ammonium acetate was added into water for sharp chromatographic peak. 0.1% glacial acetic acid with methanol-water gives a sharp peak. Hence, methanol-water containing 0.1% glacial acetic acid was selected as suitable mobile phase. Fig. 2 represents sharp peak with good symmetry and short retention time with optimized mobile phase. The reason for choosing telmisartan as IS was due to its resembling chromatographic behavior to sertraline.

### Linearity range and lower limit of quantification

The drug solution concentration ranges from 0.1-100 ng/mL was prepared to determine the range of linearity. The peak area vs. the concentration provided the calibration curve. Least-squares linear regression method (weighted, 1/x^2^) was used for the determination of linearity. The lower limit of quantification (LLOQ) represented the sensitivity of the method measured from the eight nominal drug concentrations spiked in rat plasma. The concentration below which the interday RSD exceeded 20% was taken as LLOQ. The specificity of the method was assessed with chromatographic profile of blank plasma. No interfering peak was visible and the separation of both drug and IS were achieved well with sharp peak with high resolution. The retention time of sertraline and IS were 1.25 and 1.62 min, respectively. There were no impurities observed throughout the retention time demonstrated the specificity of the technique. The linearity was in the concentration range of 0.1-100 ng/mL (r>0.987) determined using the calibration standards plot. The LLOQ value was found to be 0.1 ng/mL.

### Precision and accuracy

The method was assessed for precision and accuracy at three different concentration of drug solutions (5, 25, and 100 ng/mL). The percent relative standard deviation (RSD%) was measured at each level to validate the precision of the method in the specified limits. The RSD value within ±15% considered an appropriate limit for precision. % Accuracy was calculated as;

$$\%\;\text{accuracy}=\frac{\text{Measured}\;\text{concentration}-\text{Nominal}\;\text{concentration}\;}{\text{Nominal}\;\text{concentration}}\;X\;100$$

Accuracy below 15% considered within the acceptable limit. As shown in Table 1, the RSD% for precision were within 7.6-10.6%, and the % accuracies were ±8.0%. The results obtained were found to be within an acceptable limit. Results strongly suggested the method can offer acceptable precision and accuracy for the estimation of sertraline in biological fluids like plasma.

**Table 1:** Precision and accuracy of sertraline in rat plasma.

Matrix	QC samples	C_nominal_ (ng/mL)	Intra-day precision			Inter-day precision		
			C_measured_ (ng/mL)	% RSD	% Accuracy	C_measured_ (ng/mL)	% RSD	% Accuracy
Plasma	LQC	5	5.4 ± 0.53	9.8	8.0	4.8 ± 0.47	9.8	- 4.0
	MQC	25	24.6 ± 1.89	7.6	- 1.6	25.6 ± 2.09	8.2	2.4
	HQC	100	102.5 ± 8.62	8.4	2.5	93.2 ± 9.87	10.6	- 6.8

### Extraction recovery and matrix effect

The recovery was determined at three concentrations of QC levels (5, 25, and 100 ng/mL). The peak areas both from extracted spiked sample and post extracted spiked samples at the corresponding concentration were used to calculate the recovery (%). Comparing the peak area observed from three standard concentrations of the analyte and being spiked with plasma with that of blank samples were set to assess the matrix effect. Table 2 demonstrates the results of recovery studies. All three QC levels of sertraline stated as HQC, MQC, and LQC indicated a good % recovery. The % recovery and recovery rate of extraction was relatively stable and found within the acceptance limit (90–110%). The matrix effect of sertraline in plasma was in the range of 95.7-100.4%_,_ and 96.7% for IS, suggesting the method without a remarkable matrix effect and devoid of any ion suppression.

**Table 2:** Data showing % recovery and matrix effect.

Analyte	C_nominal_ (ng/mL)	C_measured_ (ng/mL)	% Recovery	% Matrix effect
Sertraline	5 (LQC)	4.6 ± 0.47	92 ± 1.35	100.4 ± 5.23
	25 (MQC)	24.3 ± 1.42	97.2 ± 1.86	99.7 ± 3.18
	100 (HQC)	93.6 ± 7.53	93.7 ± 1.43	95.7 ± 4.24
IS	100	88 ± 2.3	88 ± 1.03	96.7 ± 3.82

### Robustness

The chromatographic condition was changed slightly and the retention time, % recoveries, and peak area of drug and IS was determined. The chromatographic condition to assess the robustness of the method was flow rate of the mobile phase (± 100 µL mL^−1^), pH of the mobile phase (± 0.1 unit) and column temperature (± 2°C). No significant change in retention time and recoveries were observed with small change in analytical parameters representing the robustness of the method.

### Stability assessment of drug in plasma

A definite protocol was followed for stability assessment of drug in plasma. Short term stability study: storage of samples at room temperature for 24 h. Long-term stability study: storage of samples at -20°C for 28 days. Freeze-thaw stability study: three consecutive cycles of freezing at -20˚C and thawing at room temperature. Autosampler stability study: storage of sample at 4°C in the autosampler for 12 h. The results of the stability samples measured were compared with nominal values and expressed as % accuracy. The findings were within the accepted variability limits (Table 3). Neither any effect on the quantitation of plasma sample not any significant degradation was observed during stability study.

**Table 3:** Stability evaluation of the developed method of sertraline in rat plasma.

Storage conditions	C_nominal_ (ng/mL)	C_measured_ (ng/mL)	RSD (%)	% Accuracy
Short-term (24 h at room temperature)	5	4.4 ± 0.46	10.5	-12
	25	26.7 ± 1.63	6.1	6.8
	100	103.3 ± 7.53	7.3	3.3
Freeze–thaw (Three cycles)	5	5.1 ± 0.18	3.5	2
	25	24.3 ± 1.32	5.4	-2.8
	100	94.6 ± 9.86	10.4	-5.4
Autosampler for 12 h	5	5.3 ± 0.27	5.1	6
	25	25.9 ± 1.32	5.1	3.7
	100	97.8 ± 6.39	6.6	-2.2
Long-term (28 days at -20°C)	5	4.6 ± 0.13	2.8	-8
	25	23.7 ± 1.63	6.9	-5.2
	100	93.7 ± 7.56	8.1	-6.3

## Pharmacokinetic application

### Composition of lipid-based formulation

Emulsification followed by ultrasonication was used to prepare the lipid nanoparticles. Briefly, the molten solid lipid was dispersed in aqueous phase containing surfactant in water using high pressure homogenizer (silverson L4R, Buckinghamshire, UK). The o/w nanoemulsion formed was sonicated for 10 min. Rapid cooling leads to lipid crystallization and formation of lipid nanoparticles. The final composition of optimized lipid-based formulation was; 5% w/v lipid (compritol E ATO), 5% w/v surfactant (Tween 80), and 2.5% w/v sertraline.

### Animal care and handling

Approval and authorization to conduct *in vivo* pharmacokinetic study was obtained from Institutional Animal Ethics Committee. Use of experimental animals as well as care and handling were followed for the complete study period as per the guideline. Adult Sprague Dawley rats (average weight, 250 g) were used for *in vivo* experiments. Standard laboratory conditions for temperature and relative humidity were maintained for whole study period. Each cage was occupied with six animals with free access to standard laboratory diet and water.

### Dosing and collection of blood

The formulation was orally administered to each group of animals with the help of stainless-steel feeding gauge. 2.25 mg of sertraline per kg body weight was used to calculate the administered dose. The blood samples were taken from the tail vein of rat at fixed time interval and collected in microcentrifuge tubes previously added with required amount of ethylenediamine tetra acetic acid (EDTA). The tube was then centrifuged at 5,000 rpm for 20 min. The supernatant plasma was separated and stored at -21°C for analysis. Noncompartmental analysis using WinNonLin version 4.0 software was used for calculation of pharmacokinetic parameters.

### Calculation of pharmacokinetic parameters

**Fig. 3:**
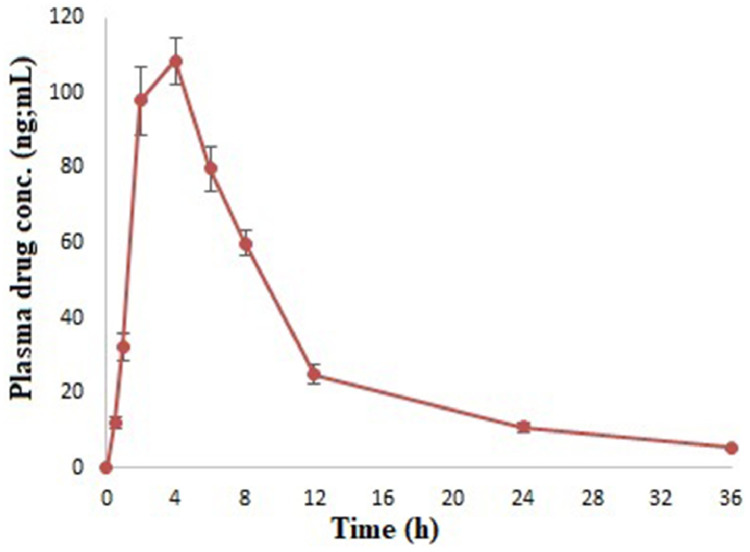
Mean plasma concentration-time curves for sertraline loaded lipid-based formulation.

The method was successfully applied to determine the concentration of sertraline in rat plasma after oral administration of formulation. Plasma concentration vs. time profile curve was used to obtain the pharmacokinetic parameters (Fig. 3). Maximum plasma drug concentration (Cmax), time to reach maximum plasma drug concentration (Tmax), Area under the curve (AUC), and maximum residence time (MRT) were calculated (Table 4). The statistical data analysis suggested no significant difference (p>0.05) in the values obtained from the developed analytical technique with that of the reference [14].

**Table 4:** Pharmacokinetic parameters following oral administration of lipid-based formulation.

Parameters	Unit	Reference	Value obtained
Cmax	ng/mL	123.69 ± 24.37	108.48 ± 17.25
Tmax	h	3.6 ± 0.3	4 ± 0.5
AUC_0 → 36_	ng/mL h	1125.67 ± 38.34	1246.74 ± 23.43
AUC_0 → ∞_	ng/mL h	1337.24 ± 23.67	1585.93 ± 27.32
MRT	h	6.58 ± 0.47	7.50 ± 0.25

## Declaration of Competing Interest

Author declares no conflict of interest.
